# Immunologic cellular characteristics of the tumour microenvironment of hepatocellular carcinoma drive patient outcomes

**DOI:** 10.1186/s12957-019-1635-3

**Published:** 2019-06-06

**Authors:** Georgi Atanasov, Karoline Dino, Katrin Schierle, Corinna Dietel, Gabriela Aust, Johann Pratschke, Daniel Seehofer, Moritz Schmelzle, Hans-Michael Hau

**Affiliations:** 1 Department of Visceral-, Transplantation-, Thoracic- and Vascular Surgery, University Hospital Leipzig, Leipzig, Germany; 2 Department of Surgery, Campus Charité Mitte Campus Virchow Klinikum, Charité – Universitätsmedizin Berlin, corporate member of Freie Universität Berlin, Humboldt-Universität zu Berlin, and Berlin Institute of Health, Berlin, Germany; 3 Berlin Institute of Health, Berlin, Germany; 4 Institute of Pathology, University Hospital Leipzig, Leipzig, Germany; 5 Department of Surgery, Research Laboratories, University of Leipzig, Leipzig, Germany

**Keywords:** Hepatocellular carcinoma, Monocytes/macrophages, Tumour-infiltrating lymphocytes, M2 macrophages, Biomarkers, Prognosis

## Abstract

**Background:**

Anti-tumour immune competence has an impact in hepatocarcinogenesis and success of anti-cancer therapies. Tumour-infiltrating lymphocytes (TILs) and monocytes/macrophages (TAMs) are proposed to have significance in cancer. However, there is only limited data concerning their impact on patient outcome and survival in hepatocellular carcinoma (HCC).

**Methods:**

Frequencies of CD68^+^, CD163^+^ M2-polarized TAMs and TILs were measured in de novo HCC tumours in non-cirrhosis (*n* = 58) using immunohistology and correlated to patients’ clinicopathological characteristics and survival rates.

**Results:**

Patients with tumours marked by appearance of TILs and CD68^+^ TAMs showed an improved 1-, 3- and 5-year recurrence-free survival (all *p* ≤ 0.05). CD68^+^ TAMs were associated with reduced incidence of recurrent and multifocal disease. Conversely, CD163^+^ TAMs were associated with multifocal HCC and lymphangiosis carcinomatosa (all *p* ≤ 0.05).

**Conclusions:**

TILs and CD68^+^ TAMs are associated with multiple tumour characteristics and patient survival in HCC. However, there is only scarce data about the biology underlying their mechanistic involvement in human tumour progression. Thus, experimental data on functional links might help develop novel immunologic checkpoint inhibitor targets for liver cancer.

## Background

Tumour-related mechanisms define the host immune functions in the tumour microenvironment and decrease the efficiency of the anti-tumour immune competence. This phenomenon plays a key role in the process of hepatocarcinogenesis, where tumour infiltration with immune competent cells exerts a strong influence on prognosis [1]. In liver cancer, tumour-associated macrophages (TAMs) and monocyte subsets with distinguished functional polarization have a significant impact on cancer-related inflammation and foster tumour escape mechanisms and progression [2]. These subpopulations of immunologic phenotypes are related to as classically (M1) or alternatively activated (M2) monocytes/macrophages. These monocytes/macrophages deploy their immunoregulatory activities in close interplay with T cell-dependent responses [2].

Tumour-infiltrating lymphocytes (TILs) play a major role in the progression of solid malignancies and can have a strong influence on the success of the related anti-cancer therapies. TILs in hepatocellular carcinoma (HCC) are mainly T cells. TILs are the hallmark of the anti-cancer immunity in human cancer, on grounds of their ability to specifically interact with and neutralize tumour-related neoantigens [3]. TILs’ immune activity may influence hepatocarcinogenesis not only by direct effects on the adaptive immune system and cytokine interactions, but also further by modulating neoangiogenesis and innate immune responses, i.e. related monocyte/macrophages functions [4–8]. Therefore, the quantification of TILs in solid tumours might help to deliver novel insights concerning their role in the process of hepatocarcinogenesis and to monitor the therapy outcome with immunologic checkpoint inhibition.

Major progress in the efficacy of adjuvant therapies for hepatic malignancies has emerged. However, the restraints of these neoadjuvant regimens constitute an important clinical problem [9]. Therefore, an additional immune checkpoint blockade represents an attractive therapeutic concept that complements these current therapies in cancer. Furthermore, the pre- and on-treatment quantification of the immunologic infiltrates could deliver novel biomarkers for state of the art disease management, based on the fact that immunological checkpoint targeting is effective only in a scarce amount of the tumour patients [10]. HCC can arise de novo in non-cirrhotic hepatic environment in approximately 20% of all cases [11]. However, this subgroup of patients usually presents at an advanced stage of hepatocarcinogenesis, due to the lack of symptoms and surveillance. Therefore, our study aimed to assess the presence and abundance of tumour-infiltrating subsets of monocytes/macrophages and TILs in advanced de novo HCC in non-cirrhosis. This study investigated also their association with tumour recurrence, patient survival and outcome.

## Methods

### Patients and tumour specimens

Our retrospective study was conducted in 58 patients with de novo HCC in non-cirrhosis. In all patients, a major liver resection with curative intent was performed. None of the included patients had history of viral hepatitis or were treated with neoadjuvant radio- and/or chemotherapy before the tumour resection. Formalin-fixed and paraffin-embedded surgical specimens, embedding a representative tumour sample for immunohistochemical staining, were obtained from the archives of the Department of Pathology. The study was conducted in accordance with the ethical guidelines of the Declaration of Helsinki and was approved by the Ethics Committee of the Leipzig University.

### Immunohistology

The protocols for immunohistology and quantification of positive staining have been published previously [12–16]. The abundance of infiltrating TILs, CD68^+^ and CD163^+^ TAMs were measured in regard to the tumour central area (TCA) and the tumour-infiltrating front (TIF). Zeiss Axio Imager A1 Phase Contrast microscope (Carl Zeiss, Jena, Germany) was used to asses positive staining. Table 1 summarizes the antibodies and reagents used to conduct immunohistology.

**Table 1:** Antibodies and reagents used for immunostaining

Antigen	m/p	Clone	Species	Company	Secondary antibody	Company	Substrate	Antigen retrieval
CD68	m	PG-M1	Mouse	Agilent	anti-mouse-Ig/ peroxidase	Vector	DAB	Tris/EDTA pH 9.0
CD163	m	10D6	Mouse	Leica Biosystems, Newcastle Upon Tyne, UK	anti-mouse-Ig/ peroxidase	Vector	DAB	10 mM citrate, pH 5.5

*DAB* 3,3′-diaminobenzidine, *m* monoclonal, *p* polyclonal, *TAM* tumor-associated macrophage

### Density quantification of cellular infiltrates

Quantification was performed as described [13]. In brief, tumour-infiltrating immune cells were categorized as negative/absent in up to 5% positive cells (0–5% positive cells, score 0) and positive/present (> 5% positive cells, score 1). HCC patients were then assigned to two groups either to be negative or positive for CD68^+^ or CD163^+^ TAMs. Evaluation of TILs was performed using routine H&E slides as described [17]. Briefly, the extent of lymphocyte abundance in the tumour area was categorized as none (score 0), low (score 1), moderate (score 2) or high (score 3). Accordingly, HCC patients were then assigned to the TIL^−^ (none to low infiltration) or TIL^+^ group (moderate to high infiltration).

### Statistical analysis

The IBM SPSS statistics software was used to perform survival and univariate analysis and to produce the Kaplan-Meier curves (Version 25/Year 2017/USA). The log-rank test was applied to compare differences in survival distributions. The Cox proportional hazards model was utilized to conduct multivariate analysis for the significant parameters in the univariate analysis. The chi-square test, Fisher’s exact test or Student’s *t* test (independent sample) were applied to compare categorical and continuous variables. Statistical differences were considered significant for *p* ≤ 0.05.

## Results

Table 2 summarizes the clinicopathological characteristics of all patients included in the current work. The studied population of HCC patients had 1-, 3- and 5-year survival of 76.4%, 64.1% and 62.2%, respectively. The recurrence-free survival rates 1-, 3- and 5- year after tumour resection were 63.5%, 57.7% and 53.6%, respectively. In 23/58 (39.7%) patients, a recurrent tumour disease was detected and 17/58 (29.3%) patients developed a local tumour recurrence. A metastatic disease was seen in 11/58 (19.0%) patients.

**Table 2:** Clinicopathological characteristics of the patients included in the study

Variable	Value (%)
No. of patients	58
Gender	
Female	13 (22.4%)
Male	45 (77.6%)
Patient age (years)	
≤ 60	21 (36.2%)
> 60	37 (63.8%)
Pathologic T stage	
T1/T2	29 (50.0%)
T3/T4	29 (50.0%)
Pathologic N stage	
Positive	11 (19.0%)
Negative	47 (81.0%)
Lymphangiosis carcinomatosa	
Positive	17 (29.3%)
Negative	41 (70.7%)
Angioinvasion	
Positive	30 (51.7%)
Negative	28 (48.3%)
Multiple tumour nodules	
With	13 (22.4%)
Without	45 (77.6%)
Tumor size (mm)	
≤ 50	11 (19.0%)
> 50	47 (81.0%)
Pathologic R category	
R0	49 (84.5%)
R1/R2	9 (15.5%)
Histologic differentiation	
Well	11 (19.0%)
Moderate/poor	47 (81.0%)
Distant metastases	
With	11 (19.0%)
Without	47 (81.0%)
Tumor recurrence	
With	23 (39.7%)
Without	35 (60.3%)
Local recurrence	
With	17 (29.3%)
Without	41 (70.7%)

### Distribution of monocytes/macrophages and tumour-infiltrating lymphocytes in HCC

**Fig. 1:**
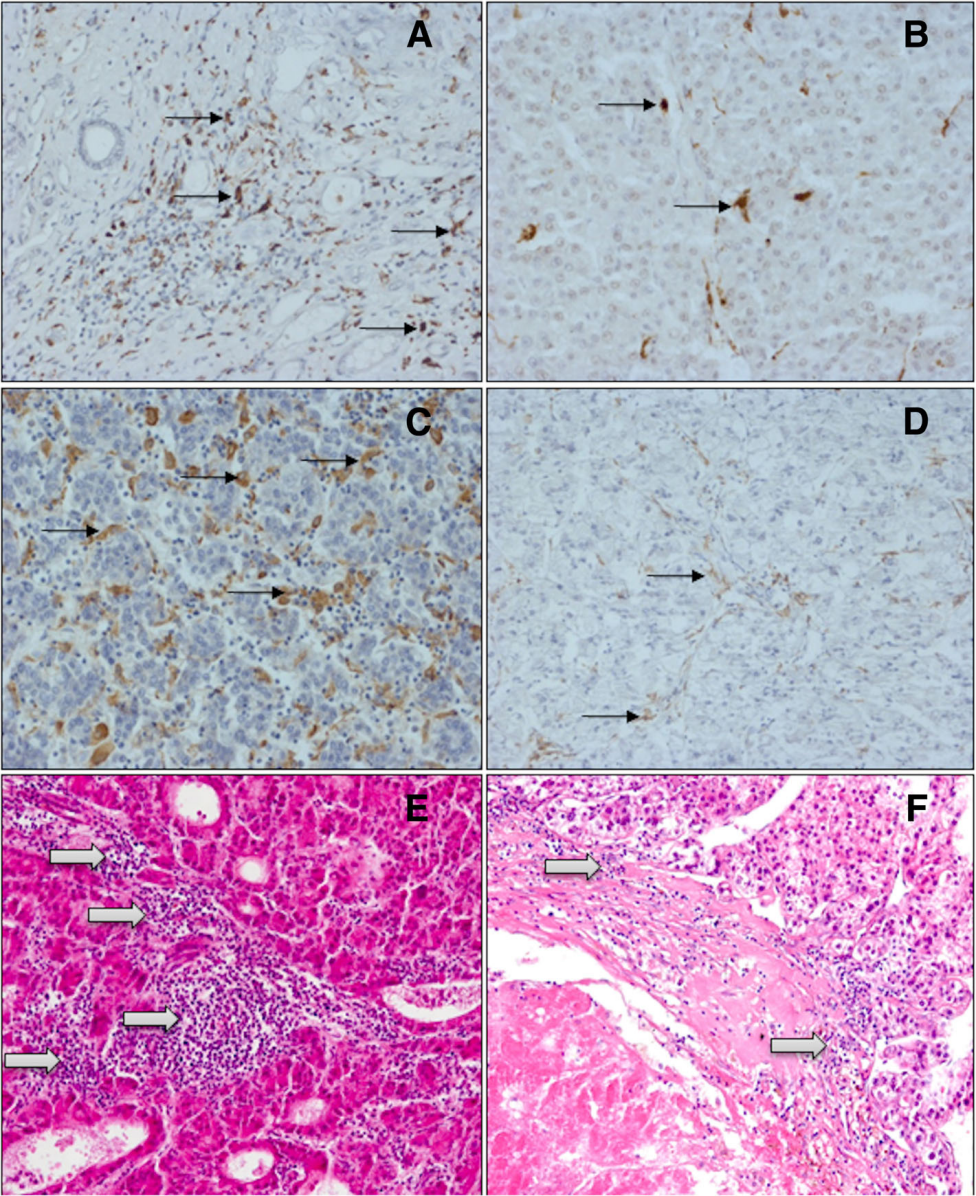
Immunohistology of monocytes/macrophages and H&E staining of TILs in tumour central area (TCA) of HCC specimens. **a** High density of CD68^+^ TAMs. **b** Low density of CD68^+^ TAMs. **c** High density of CD163^+^ TAMs. **d** Low density of CD163^+^ TAMs. **e** High density of TILs. **f** Low density of TILs. Legend: left column, high density; right column, low density. Black arrows indicate monocytes/macrophages, white arrows TILs

Figure 1 provides characteristic images for the abundance of TILs and CD68^+^ or CD163^+^ TAMs in HCC. Tables 2, 3, 4 and 5 summarize the respective statistical data of the patients. TILs and CD68^+^ or CD163^+^ TAMs revealed a homogeneous expression pattern in TCA and TIF and were also detected in areas of HCC necrosis (Fig. 1a–f).

**Table 3:** Correlation of CD68^+^ TAMs at the tumour-infiltrating front (TIF) with clinicopathological characteristics in HCC

Variable	CD68^+^/TIF	CD68^−^/TIF	*p* value
No. of patients	53	5	
Patient age, years			0.324
≤ 60	33 (62.3%)	4 (80.0%)	
> 60	20 (37.7%)	1 (20.0%)	
Gender			0.430
Female	42 (79.2%)	3 (60.0%)	
Male	11 (20.8%)	2 (40.0%)	
Local tumour recurrence			0.583
Positive	15 (28.3%)	2 (40.0%)	
Negative	38 (71.7%)	3 (60.0%)	
Overall tumour recurrence			0.050
Positive	19 (35.8%)	4 (80.0%)	
Negative	34 (64.2%)	1 (20.0%)	
Distant Metastases			0.209
Positive	9 (17.0%)	2 (40.0%)	
Negative	44 (83.0%)	3 (60.0%)	
Multiple tumour nodules			0.035
Positive	10 (18.9%)	2 (40.0%)	
Negative	43 (81.1%)	3 (60.0%)	
Tumour size (mm)			0.951
≤ 50	10 (10.0%)	1 (20.0%)	
> 50	43 (90.0%)	4 (80.0%)	
R status			0.772
Positive	8 (15.1%)	1 (20.0%)	
Negative	45 (84.9%)	4 (80.0%)	
Angioinvasion			0.583
Positive	25 (47.2%)	2 (40.0%)	
Negative	28 (52.8%)	3 (60.0%)	
Lymphangiosis carcinomatosa			0.583
Positive	15 (28.3%)	2 (40.0%)	
Negative	38 (71.8%)	3 (60.0%)	
Histologic differentiation			0.951
Well	10 (10.0%)		1 (20.0%)
Moderate/poor	43 (90.0%)		4 (80.0%)
Pathologic T stage			0.148
T1/T2	28 (53.8%)	1 (20.0%)	
T3/T4	24 (46.2%)	4 (80.0%)	
Pathologic N stage			0.658
Positive	2 (03.8%)	0 (00.0%)	
Negative	51 (96.2%)	5 (100.0%)

**Table 4:** Correlation CD163^+^ TAMs in the tumour central area (TCA) or tumour-infiltrating front (TIF) with clinicopathological characteristics in HCC

Variable	CD163^+^/TCA	CD163^−^/TCA	*p* value
No. of patients	42	16	
Patient age, years			0.177
≤ 60	13 (31.0%)	8 (50.0%)	
> 60	29 (69.9%)	8 (50.0%)	
Gender			0.680
Female	10 (76.2%)	3 (18.8%)	
Male	32 (23.8%)	13 (81.2%)	
Local tumour recurrence			0.398
Positive	11 (26.2%)	6 (37.5%)	
Negative	31 (73.8%)	10 (62.5%)	
Overall tumour recurrence			0.320
Positive	15 (35.7%)	8 (50.0%)	
Negative	27 (64.3%)	8 (50.0%)	
Distant metastases			0.469
Positive	7 (16.7%)	4 (25.0%)	
Negative	35 (83.3%)	12 (75.0%)	
Multiple tumour nodules			0.016
Positive	36 (85.7%)	9 (56.3%)	
Negative	6 (14.3%)	7 (43.8%)	
Tumour size (mm)			0.438
≤ 50	9 (21.4%)	2 (12.5%)	
> 50	33 (78.6%)	14 (87.5%)	
R status			0.695
Positive	35 (15.1%)	2 (12.5%)	
Negative	7 (84.9%)	14 (87.5%)	
Angioinvasion			0.311
Positive	20 (47.6%)	10 (62.5%)	
Negative	22 (52.4%)	6 (37.5%)	
Lymphangiosis carcinomatosa			0.656
Positive	13 (31.0%)	4 (25.0%)	
Negative	29 (69.0%)	12 (75.0%)	
Histologic differentiation			0.979
Well	8 (19.0%)	3 (18.8%)	
Moderate/poor	34 (81.0%)	13 (81.3%)	
Pathologic T stage			0.501
T1/T2	22 (53.7%)	7 (43.8%)	
T3/T4	19 (46.3%)	9 (56.3%)	
Pathologic N stage			0.470
Positive	1 (02.4%)	1 (06.3%)	
Negative	41 (97.6%)	15 (93.7%)	
No. of patients	19	39	
Lymphangiosis carcinomatosa			0.035
Positive	9 (47.4%)	8 (20.5%)	
Negative	10 (52.6%)	31 (79.5%)

**Table 5:** Correlation of TILs in the tumour central area (TCA) with clinicopathological characteristics in HCC

Variable	TIL^+^/TCA	TIL^−^/TCA	*p* value
No. of patients	20	38	
Patient age, years			0.476
≤ 60	6 (30.0%)	15 (39.1%)	
> 60	14 (70.0%)	23 (60.9%)	
Gender			0.732
Female	15 (75.0%)	30 (78.9%)	
Male	5 (25.0%)	8 (21.1%)	
Local tumour recurrence			0.933
Positive	6 (30.0%)	11 (28.9%)	
Negative	14 (70.0%)	27 (71.1%)	
Overall tumour recurrence			0.546
Positive	9 (45.0%)	14 (36.8%)	
Negative	11 (55.0%)	24 (63.2%)	
Metastases			0.884
Positive	4 (20.0%)	7 (18.4%)	
Negative	16 (80.0%)	31 (81.6%)	
Multiple tumour nodules			0.100
Positive	18 (90.0%)	27 (71.1%)	
Negative	2 (10.0%)	11 (28.9%)	
Tumour size (mm)			0.206
≤ 50	2 (10.0%)	9 (23.7%)	
> 50	18 (90.0%)	29 (76.3%)	
R status			0.148
Positive	15 (75.0%)	34 (89.5%)	
Negative	5 (25.0%)	4 (10.5%)	
Angioinvasion			0.717
Positive	11 (55.0%)	19 (50.0%)	
Negative	9 (45.0%)	19 (50.0%)	
Lymphangiosis carcinomatosa			0.057
Positive	9 (45.0%)	30 (78.9%)	
Negative	11 (45.0%)	8 (21.1%)	
Histologic differentiation			0.576
Well	3 (15.0%)	8 (21.1%)	
Moderate/poor	17 (85.0%)	30 (78.9%)	
Pathologic T stage			0.708
T1/T2	9 (47.4%)	20 (52.6%)	
T3/T4	10 (52.6%)	18 (47.4%)	
Pathologic N stage			0.296
Positive	0 (00.0%)	2 (5.3%)	
Negative	20 (100.0%)	36 (94.7%)	
CD68^+^ TAMs/TCA			0.008
Positive	20 (100.0%)	11 (28.9%)	
Negative	0 (00.0%)	27 (71.1%)	

### Monocytes/macrophages are associated with reduced incidence of tumour recurrence and formation of multiple tumour nodules in HCC patients

CD68^+^ TAMs in TIF were associated with reduced occurrence of recurrent HCC. In the CD68^+^ group, only 19/53 (35.8%) patients suffered overall tumour recurrence, whereas in the CD68^−^ group, 4/5 (80.0%) patients had recurrent disease (*p* = 0.05; Table 3). CD68^+^ TAMs in TIF were also correlated with reduced formation of multiple tumour nodules (*p* = 0.035). In the CD68^+^ group, only 10/53 (18.9%) patients showed this feature, whereas in the CD68^−^ group, these were 2/5 (40.0%) patients (*p* = 0.035).

### M2-polarized macrophages are associated with lymphangiosis carcinomatosa and formation of multiple tumour nodules in HCC patients

CD163^+^ TAMs in TCA were associated with the formation of multiple tumour nodules (*p* = 0.016; Table 4). In the CD163^+^ group, 36/42 (85.7%) patients had multiple tumour nodules; in the CD163^−^ group in 9/16 (56.3%) patients, this was diagnosed (*p* = 0.016). Moreover, when considering the TIF, in the CD163^−^ group, 31/39 (79.5%) patients had absence of lymphangiosis carcinomatosa. In the CD163^+^ group, these were 10/19 (52.6%) patients (*p* = 0.035). No significant association between CD163^+^ TAMs in TCA or TIF with CD68^+^ TAMs could be detected.

### Tumour-infiltrating lymphocytes are associated with intratumoural monocytes/macrophages in HCC patients

TILs in TCA or TIF were not correlated with clinicopathological features of HCC patients (Table 5). However, in regard to the TCA, TILs and CD68^+^ TAMs revealed a strong correlation. In the TIL^+^ group in 20/20 (100%) and in the TIL^−^ group in only 11/38 (28.9%) patients, high frequencies of CD68^+^ TAMs were detected (*p* = 0.008). No significant correlations between TILs in TCA or TIF with CD163^+^ TAMs could be observed.

### Influence of monocytes/macrophages and tumour-infiltrating lymphocytes on survival in HCC patients

**Fig. 2:**
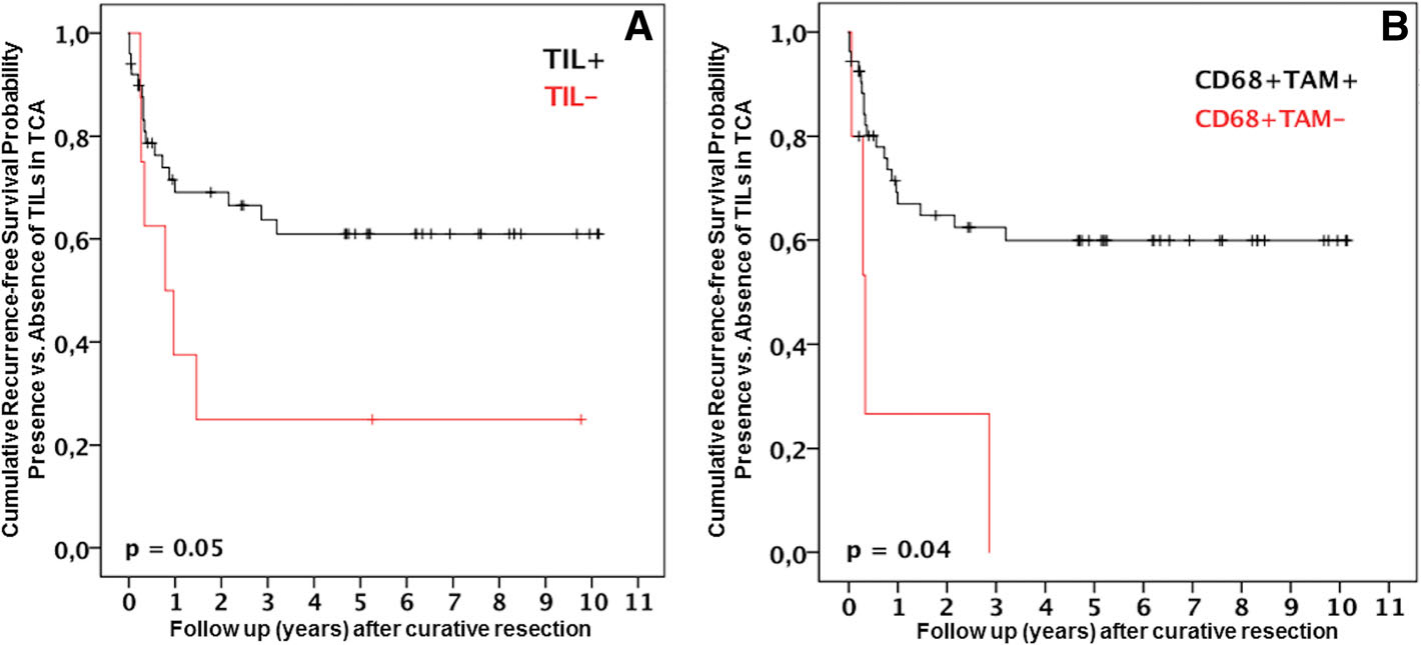
**a** Recurrence-free survival for TILs in the TCA. TIL^−^ refers to the TIL^-^ group. TIL+ refers to the TIL^+^ group. **b** Recurrence-free survival for CD68^+^ TAMs in the TIF. CD68^+^ TAM^−^ refers to the CD68^−^ group. CD68^+^ TAM^+^ refers to the CD68^+^ group.

In our study, CD68^+^ TAMs and TILs were associated with patients’ recurrence-free survival. Figure 2 shows the Kaplan-Meier survival curves. Tables 2, 3, 4 and 5 show the statistical evaluation of all patients. Recurrence-free survival rates were significantly improved in patients with TILs in TCA (Fig. 2a). One, 3 and 5 years after surgery, these were 68.9%, 63.9% and 61.6%, respectively. Conversely, the survival was 37.8%, 23.4% and 23.4% at 1, 3 and 5 years post-surgery, respectively, in patients without TILs in TCA (*p* = 0.05). Similar data was obtained in regard to CD68^+^ TAMs in TIF (Fig. 2b). The recurrence-free survival rates were 66.9%, 63.3% and 60.0% at 1, 3 and 5 years for patients with CD68^+^ TAMs in TIF. Contrarily, the recurrence-free survival was 28.7% at 1 year post-surgery in HCC patients without these cells in the TIF. Of note, survival beyond 3 years after surgery could not be reached in patients without CD68^+^ TAMs in TIF (*p* = 0.04). CD163^+^ TAMs in TCA or TIF did not reveal any significant correlation with overall or recurrence-free survival of the HCC patients (CD163^+^ TAMs in TCA: overall survival *p* = 0.858, recurrence-free survival *p* = 0.283; CD163^+^ TAMs in TIF: overall survival *p* = 0.410, recurrence-free survival *p* = 0.405).

## Discussion

In the present work, we determined the level of abundance of monocytes/macrophages and TILs in tumour specimens of patients with de novo HCC in non-cirrhosis after oncologic resection. In addition, their association with patient’ clinicopathologic characteristics and survival was analysed. The major discoveries were (1) CD68^+^ TAMs were associated with decreased rates of recurrent and multifocal disease; (2) conversely, M2-polarized TAMs correlated with lymphangiosis carcinomatosa and multifocal HCC; (3) TILs and infiltrating CD68^+^ TAMs were strongly associated in HCC; and (4) had a potent influence on recurrence-free survival.

In this study, we demonstrated that CD68^+^ TAMs and M2-polarized TAMs correlate with established clinicopathologic features of advanced de novo HCC in non-cirrhosis. Of note, 29/58 (50%) of the HCC patients in our study had T3/T4 tumours and 47/58 (81%) tumours exhibited moderate/poor histologic differentiation. These results are in accordance with previously published results in HCC and other hepatobiliary tumours, which report on a negative impact of M2 polarization state of infiltrating TAMs on patient survival and outcome [18, 19]. However, when considering CD68^+^ TAMs, most published results demonstrate a negative value in regard to patient outcome [20]. Here, CD68^+^ TAMs were associated with significantly prolonged survival. A possible scenario for our results could be that TAMs comprise a diverse and heterogeneous cell population which can express markers typical not only for M1 or M2 polarization states. Their functionality strongly depends on the signals deployed by the tumour microenvironment, i.e. TAMs re-education and reprogramming as classical tumour escape mechanisms. In line with this, Li et al. demonstrated CD68^+^ TAMs to be also CD204^+^ or CD169^+^ cells. The tissue frequency of CD204^+^ TAMs associated with poor outcome. Conversely, CD169^+^ TAMs were associated with better survival [19]. Insofar, additional research is needed to investigate the mechanistic interplay between diverse TAMs subpopulations and the tumour microenvironment.

The predominant immune cell population in the tumour vicinity consists mainly of TILs. Accumulating scientific data demonstrates that the type, density and localization of host immunologic tumour infiltration influence its malignant behaviour and could provide clinically informative prognostic biomarkers [21–23]. Furthermore, this immunologic reaction could characterize patient outcome to a greater extent than the diagnostics for the staging of cancer, which are currently applied by conventional histopathology [24]. Consequently, TILs were identified as a reliable immunologic tool in the tumour microenvironment that could be used in clinical trials and translational research. Insofar, here we focused on the prognostic capacity of TILs in tumour specimens of HCC. Our findings indicate that the presence of TILs significantly influence survival of HCC patients after curative surgery. Of note, in our work, intratumoural prevalence of TILs in liver cancer was correlated with a high frequency of invading TAMs. Thus, it appears possible that infiltrating hepatic monocytes/macrophages and TILs comprise a coherent immunological construct that exerts a significant impact in the process of hepatocarcinogenesis.

In our work, in the setting of oncologic HCC resection, TILs and CD68+ TAMs were associated with better survival rates, which is in line with most published data about TILs’ importance in solid cancer that delineates their presence or high tumour density to be associated with improved patient survival [17, 25–27]. Insofar, in this study, we suggest TILs and CD68^+^ TAMs to be reliable cancer biomarkers prognosticating survival and outcome of HCC patients after surgery. However, a possible limitation of the current work is the descriptive nature of our results and the small number of HCC patients with de novo HCC in non-cirrhosis.

## Conclusions

Our study demonstrates that TILs, infiltrating monocytes/macrophages and their functional polarization state associate with multiple tumour characteristics and patient survival in HCC. However, there is only scarce data about the biology underlying mechanistic involvement of TILs, monocytes/macrophages and their polarization in M1 or M2 subtypes in human tumour progression. Thus, a further examination of underlying functional mechanisms that might help develop novel immunologic checkpoint inhibitor targets for liver cancer is warranted.

## Data Availability

The datasets used and/or analysed during the current study are available from the corresponding author on reasonable request.

## Abbreviations

CCA: Cholangiocarcinoma
HCC: Hepatocellular carcinoma
PDAC: Periductal adenocarcinoma of the pancreas
TAMs: Tumour-associated macrophages
TCA: Tumour central area
TIF: Tumour-infiltrating front
